# Non-Enzymatic Electrochemical Sensing of Malathion Pesticide in Tomato and Apple Samples Based on Gold Nanoparticles-Chitosan-Ionic Liquid Hybrid Nanocomposite

**DOI:** 10.3390/s18030773

**Published:** 2018-03-05

**Authors:** Gulcin Bolat, Serdar Abaci

**Affiliations:** Department of Chemistry, Faculty of Science, Hacettepe University, 06800 Beytepe-Ankara, Turkey; gbolat@hacettepe.edu.tr

**Keywords:** malathion, gold nanoparticles, ionic liquids, organophosphorus (OP) pesticides, food safety, electrochemical sensor, direct electrochemical detection

## Abstract

Malathion (MLT) is an organophosphorous type pesticide and having seriously high toxicity and electrochemical platforms for rapid, simple, inexpensive and sensitive determination of pesticides is still a special concern. This paper describes a simple preparation of a composite film consisting of ionic liquid (IL), chitosan (CS) and electrochemically synthesized gold nanoparticles (AuNPs) on single use pencil graphite electrodes (PGEs). The microscopic and electrochemical characterization of AuNP-CS-IL/PGE was studied using scanning electron microscopy, cyclic voltammetry and electrochemical impedance spectroscopy. This fabricated surface was then explored for the first time as a sensing matrix for the non-enzymatic electrochemical sensing of malathion by cyclic voltammetry and square wave voltammetry measurements. The proposed AuNP-CS-IL/PGE showed excellent characteristics and possessed remarkable affinity for malathion. The voltammetric current response exhibited two linear dynamic ranges, 0.89–5.94 nM and 5.94–44.6 nM reflecting two binding sites, with a detection limit of 0.68 nM. The method was applied in real sample analysis of apple and tomato. The results demonstrate the feasibility of AuNP-CS-IL-modified electrodes for simple, fast, ultrasensitive and inexpensive detection of MLT.

## 1. Introduction

Organophosphorus pesticides (OPs) are an integral part of modern agriculture due to their high insecticidal activity against pests that are especially corruptive to crops and foods [1]. OPs are widely applied all over the world to increase the agricultural productivity. They can exert irreversible inhibitory effect towards the acetylcholinesterase (AChE) enzyme, which is vital for the functioning of the central nervous system in both humans and insects [2]. Over-exposure of these neurotoxic compounds and also their metabolites to non-target organisms (humans, fish and birds) rather than target species have resulted in serious health problems both in humans and the other living organisms. The extensive utilization and bioaccumulation of these compounds in soil and water has led to significant environmental pollution and ecological problems. Owing to their existence in water and food, they may cause long-term damage to human health.

The title compound malathion (*S-bis* (etoxycarbonyl) ethyl *O*,*O*-dimetyl phosphorodithioate), is a broad spectrum, nonsystemic insecticide belonging to the OP class. It is extensively used in public health programs against mosquito-borne illness, fruit fly eradication programs and indoor and outdoor residentals [3]. Malathion (MLT) is also included in some special shampoos for controlling lice. It is highly toxic to aquatic organisms and honey bees, and is moderately toxic and neurotoxic to humans. It has been also shown that malathion is mutagenic to mammals and could be adsorbed by the body through skin, ingestion or inhalation [3,4].

Therefore, it is necessary to establish sensitive and accurate methods for the determination of these toxic agents. The quantitative detection of malathion has been carried out by several methods including colorimetry [5], Fourier transform–Raman (FT–Raman) spectrometry [6], capillary electrophoresis [7], high performance liquid chromagraphy [8] and gas chromatography coupled with a flame ionisation detector [9].

Many of the above-mentioned methods are sensitive, reliable and efficient, but they have limitations such as requiring expensive, bulky instrumentation, complicated operational and pretreatment processes, high volumes of toxic reagents and long analysis times. On the other hand, electrochemical biosensors are widely used because of their high sensitivity, good selectivity, rapid response times, simpleness, ease of preparation and low cost. Electrochemical methods are also amenable to on-site monitoring of the analytes and miniaturization. Most of the studies focused on the electrochemical detection of malathion using acetylcholinesterase enzyme, AChE, biosensors functioning based on the irreversible inactivation of enzymatic activity. These AChE based electrochemical biosensors were constructed using thiophene derivative conducting polymer [10], reduced graphene oxide-Au nanoparticles-β-cyclodextrin/Prussian blue-chitosan nanocomposite [11], Fe_3_O_4_ nanoparticles and poly(indole-5-carboxylic acid) [12] and silica sol gel [4]. However, immobilization of enzymes for the construction of biosensors has some strains in applications due to high cost, denaturation, sensitivity to operational conditions and instability of enzymes [13,14]. Recently, only a few studies have been reported for the enzymeless electrochemical detection of malathion. In these systems, electrochemical signal change was monitored after the interaction of malathion with the electroactive material on the sensor surface. In this concept, CuO nanowires–SWCNTs hybrid [15], amino acid assisted growth of CuO nanostructures [16], and polyaniline nanofibers/single wall carbon nanotubes composite film [3] were used as indicators. In addition, alternative recognition elements are still needed in order to perform fast, simple and sensitive non-enzymatic analysis of malathion.

Room temperature ionic liquids (ILs) with high ionic conductivity, good stability and biocompability provide remarkable increase in the electrochemical response of the sensors [17]. Chitosan (CS) is a functional material that shows good film-forming ability, good adhesion and biocompatibility properties, which are favourable for biosensing platforms. Moreover, functionalization of the electrode surfaces with gold nanoparticles (AuNPs) provides high electrocatalytic activity and surface area, good electronic conductivity and biocompability in sensor applications [18]. In addition, it is well known that thio-containing compounds (like in malathion) bind to Au containing surfaces with high affinity by formation of Au-S bonds. Combining these important features of CS, IL and AuNPs to modify electrode surfaces appears advantageous as they offer great potential to be exploited as a sensing platform for MLT detection.

The electrode material is crucial for electrochemical sensors in terms of selectivity, stability, cost and sensitivity of the fabricated sensor. As a class of disposable electrodes, pencil graphite electrodes (PGEs) bring some important advantages in electrochemical sensing studies due to their cheapness, low technology requirements, commercial availability, chemical inertness and high electrochemical reactivity [19]. Moreover, PGE surfaces provide ability to work in a wide potential window, ease of modification/functionalization and surface renewal without any need for polishing. They also serve as substrates for enhanced adsorption of analytes. Consequently, they have been utilized as an alternative to other carbon based electrodes in recent studies [20].

To the best of our knowledge, there are no reports on non-enzymatic and direct detection of malathion based on gold nanoparticles-chitosan-hydrophilic ionic liquid PGEs. In the present work, the major aim is to explore the advantages of combining AuNPs, CS and IL for the modification of PGE to get enhanced and direct electrochemical signals for MLT. Integration of the unique properties of these functional materials as a sensing interface has led to develop a sensitive electrochemical sensor for MLT detection. The electrochemical sensor named as AuNP-CS-IL-modified PGE showed a low detection limit and wide linear range. The proposed non-enzymatic approach was simple, efficient and cost-effective. The established method was successfully employed for real sample analysis of MLT in tomato and apple samples.

## 2. Materials and Methods

### 2.1. Apparatus

All the electrochemical measurements (CV, DPV and EIS curves) were carried out using CHI 660 C Electrochemical workstation (CH Instrument, Austin, TX, USA). A three-electrode cell was comprised of a pencil graphite electrode, PGE, as a working electrode a platinum wire as a counter electrode and an Ag/AgCl/3.0 M KCl as a reference electrode. A flow of N_2_ gas was maintained over the electrolyte during the electrochemical measurements. A rotring pencil was designed as a working electrode and used to hold graphite leads (degree HB, 0.5 mm) in which the electrical contact was achieved by soldering a copper wire to the metallic holder of the pencil. The SEM micrographs were obtained with Quanta FEG 200 Model E-SEM (FEI Company World Headquarters, OR, USA).

### 2.2. Chemicals and Reagents

Chitosan, HAuCl_4_·3H_2_O, 1-butyl-3-methylimidazolium tetrafluorophosphate, (BMIMPF_6_) ≥ 97.0% and malathion pesticide were purchased from Sigma-Aldrich (St. Louis, MO, USA). Acetic acid, boric acid, phosphoric acid were supplied by Merck (St. Louis, MO, USA). Britton–Robinson buffer solution (BR) was prepared from a mixture of phosphoric acid, acetic acid, and boric acid, each component at 40 mM and required pH values were adjusted with sodium hydroxide (0.2 M). A 0.745 mM stock solution of malathion was prepared in ethanol and diluted with an appropriate amount of Britton–Robinson buffer solution to obtain malathion solutions in the working concentration range. K_4_Fe(CN)_6_ from AnalaR Analytical Reagents (VWR International Ltd., Lutterworth, UK), K_3_Fe(CN)_6_ from the Fischer Scientific Company (Hampton, NH, USA) and KCl from Merck (Kenilworth, NJ, USA) were used to prepare redox solution for CV and EIS characterizations. All the other chemicals were of analytical reagent grade.

### 2.3. Preparation of Modified Electrodes

**Scheme 1:**
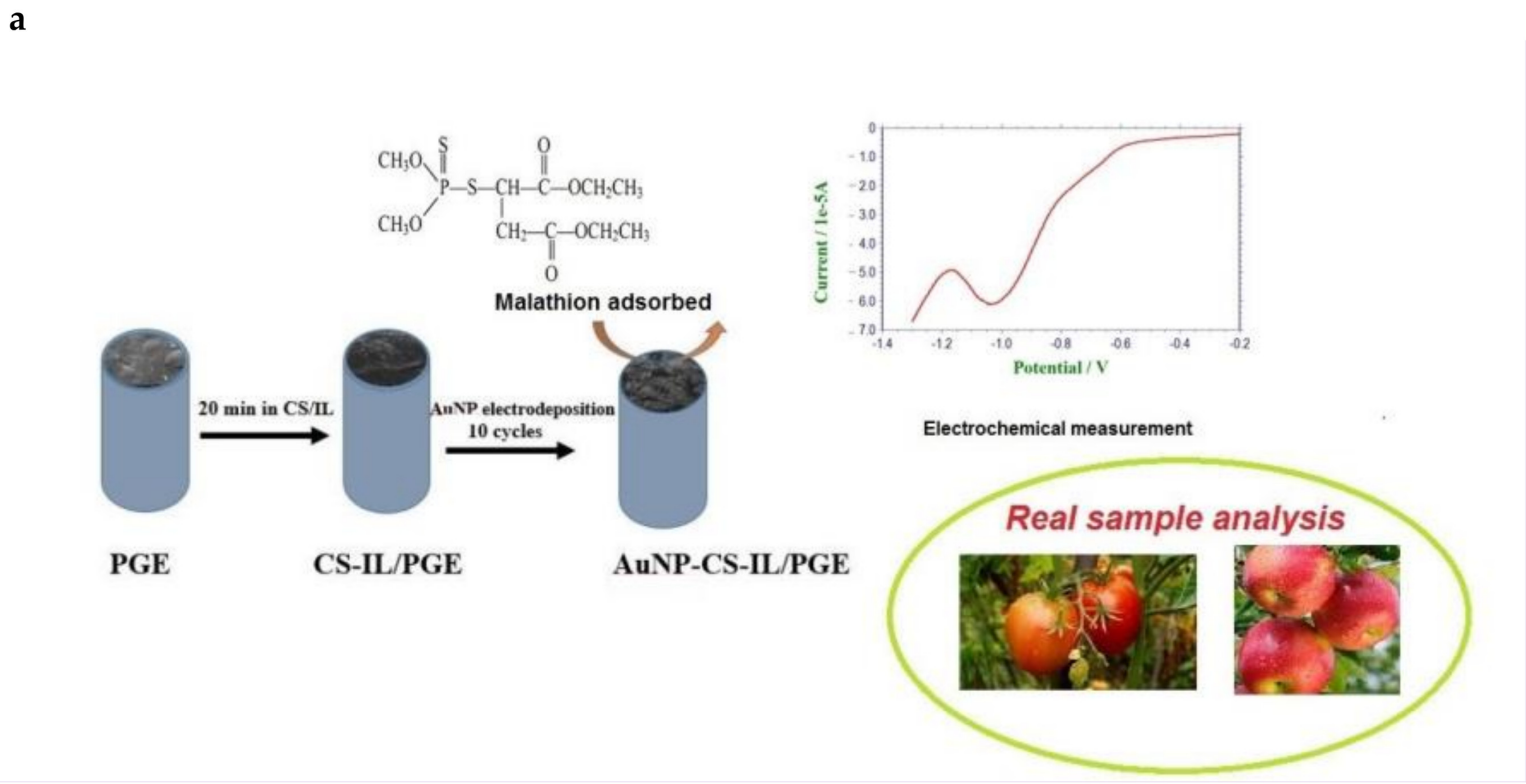
The fabrication of the AuNP-CS-IL/PGE and malathion sensing on the nano-electrode.

The nanocomposites were prepared by two steps, as follows: first, chitosan solution (0.5%) was prepared by dissolving 0.50 g of chitosan in 100 mL 1.0% (*v/v*) acetic acid. In addition, 300 µL of BMIMPF_6_, IL was added into 100 µL chitosan solution (0.5%) and mixed by agitating ultrasonically. A uniform CS-IL film was coated on PGE was obtained by immersing 0.5 cm of pencil leads in this solution for 20 min and allowed to dry at room temperature. Subsequently, electrodeposition of AuNPs on CS/IL/PGE was carried out in aqueous solution containing 0.5 mM of HAuCl_4_·3H_2_O in the presence of 0.1 M KCl and the potential was scanned at 0.1 V s^−1^ for 10 cycles in the range of 0.2–0.8 V vs. Ag/AgCl. The electrode was then rinsed with water and denoted as AuNP-CS-IL/PGE. All measurements were performed with a freshly prepared new surface. The fabrication process is depicted in Scheme 1.

### 2.4. Real Samples

The tomato and apple samples were obtained from the local market (Ankara, Turkey), cleaned with distilled water, and chopped into small pieces. In addition, 10 g of each chopped samples were added into 10 mL ether for the extraction for 2 h. The extract was taken and filtered through a 0.45-µm membrane then evaporated to dryness. Furthermore, 2.0 mL of ethanol was added to dissolve the filtrate and diluted to 100 mL with BR buffer. The samples were then spiked with a known concentration of MLT and used immediately for analysis.

## 3. Results and Discussion

### 3.1. Morphological and Electrochemical Characterization of AuNP-CS-IL/PGE

**Figure 1:**
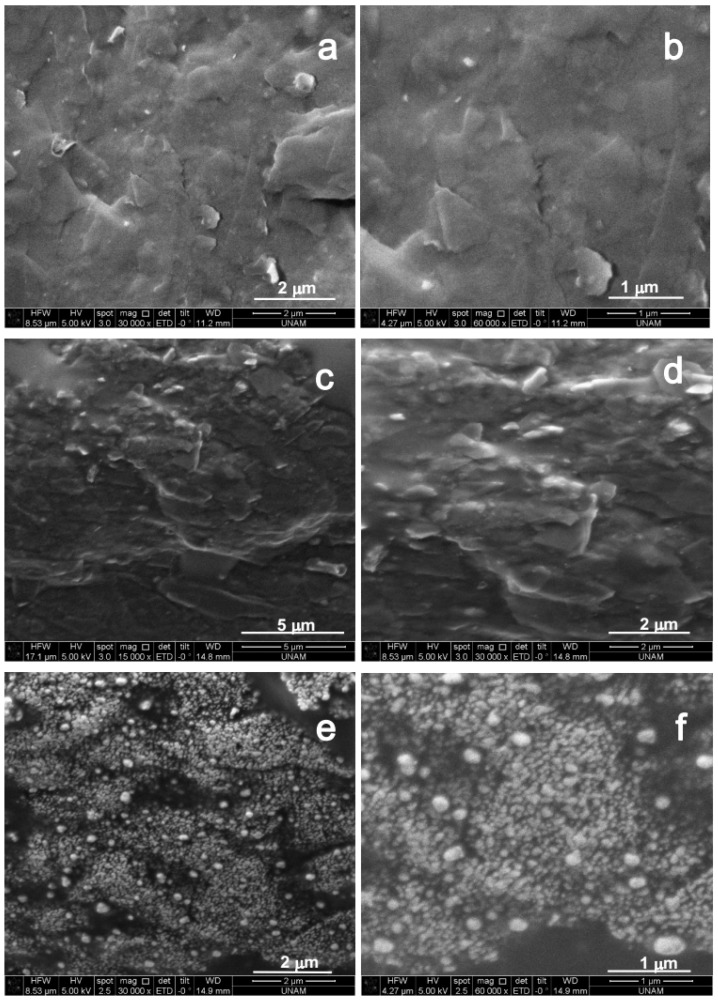
SEM images for (**a**,**b**) bare PGE, (**c**,**d**) CS-IL/PGE and (**e**,**f**) AuNP-CS-IL/PGE (30,000 and 60,000 fold magnitudes).

Figure 1 illustrates the scanning electron microscopy (SEM) images for step-by-step modification of the electrode in two different magnitudes. Bare PGE displayed a slightly uneven structure due to the separated layers of graphite flakes (Figure 1a,b). A more uniform surface with gel like rough structure obtained after the introduction of CS-IL on the PGE surface, due to the penetration of viscous CS polymer and ionic liquid into graphite layers (Figure 1c,d). IL acted as a binder in the structure. In addition, it can be observed that the conductive structure of encapsulated IL has caused the surface to appear brighter [21]. Figure 1e,f confirmed that AuNPs in globular shape were electrodeposited on the CS-IL/PGE successfully. The average diameter of the particles was about 50 nm. Interaction between the negatively charged AuCl_4_^−^ ions and the positively charged chitosan resulted in well-ordered nanoparticle formation. Furthermore, the agglomeration of nanoparticles was blocked by the amine groups of chitosan. This result agrees with the previous studies [22,23]. Additionally, the small size of gold nanoparticles was attributed to the enhanced nucleation rate due to the low surface tension of IL in the composite matrix.

**Figure 2:**
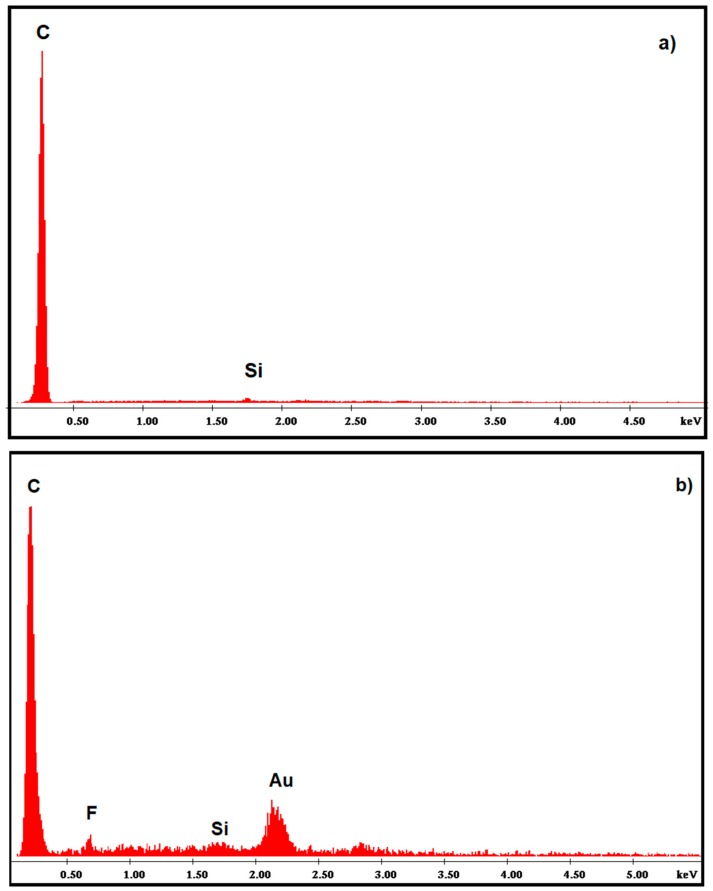
EDS of (**a**) bare PGE (**b**) AuNP-CS-IL/PGE.

Energy dispersive X-ray spectrometry (EDS) analysis results also confirmed the presence of gold on the graphite surface, which primarily consisted of carbon and silicon. Fluoride content found in the composite may be due to the ionic liquid, 1-butyl-3-methylimidazolium hexafluorophosphate (Figure 2a,b). Based on these studies, the obtained films were shown to have three-dimensional structure and enhanced surface area, providing the opportunity to be useful in sensing applications.

**Figure 3:**
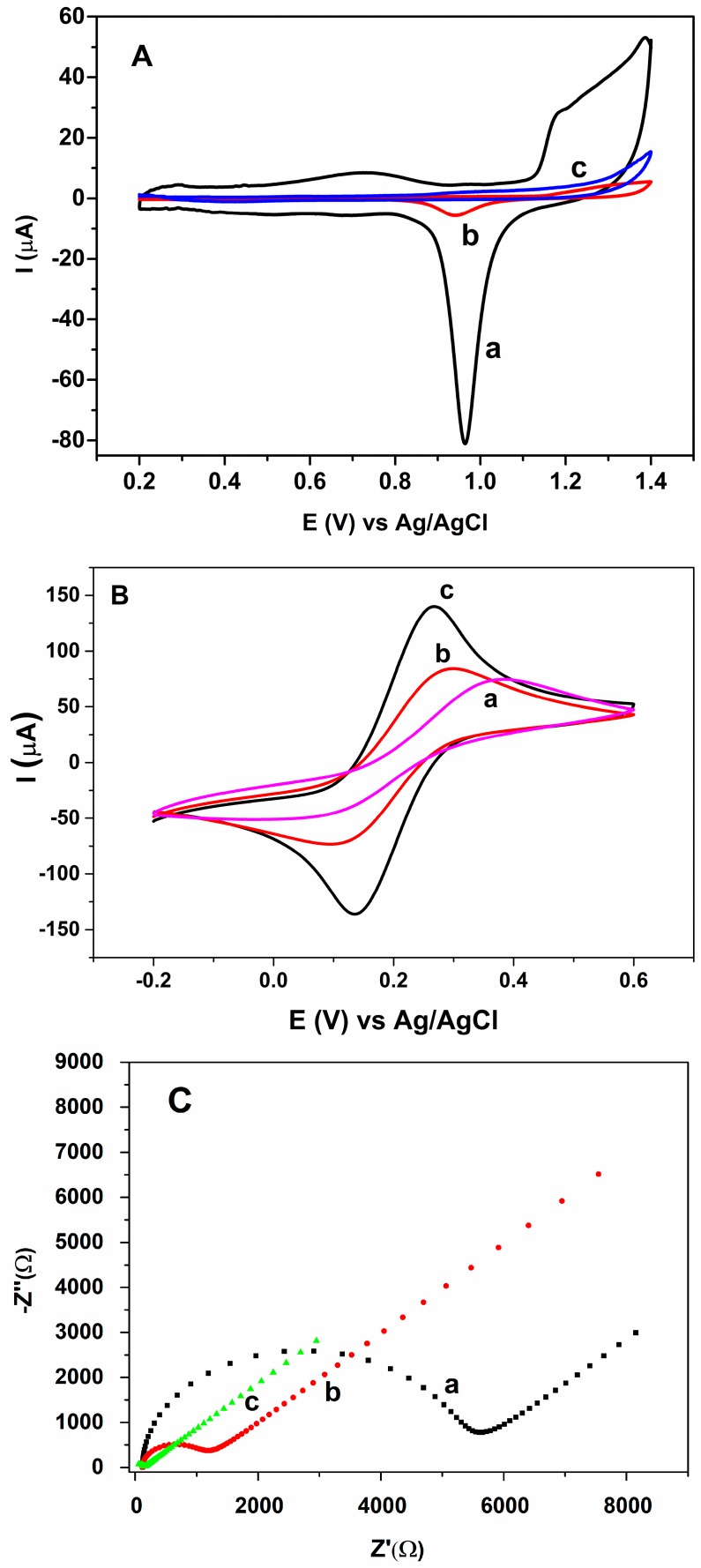
(**A**) cyclic voltammograms in 0.5 M H_2_SO_4_ for (a) AuNP-CS-IL/PGE (b) bare Au disc electrode (c) bare PGE. Scan rate: 50 mV s^−1^; (**B**) CVs of (a) Bare PGE (b) CS-IL/PGE (c) AuNP-CS-IL/PGE in 0.1 M KCl containing 5.0 mM [Fe(CN)_6_]^3−/4−^. Scan rate: 50 mV s^−1^; (**C**) electrochemical impedance spectroscopy (EIS) of (a) Bare PGE (b) CS-IL/PGE (c) AuNP-CS-IL/PGE in 0.1 M KCl containing 5.0 mM [Fe(CN)_6_]^3−/4−^ (frequency range 0.1–100,000 Hz).

AuNPs electrodeposited on the CS-IL/PGE were characterized in 0.5 M H_2_SO_4_ (N_2_ saturated) in the potential range 0.20 V and 1.40 V by cyclic voltammetry (Figure 3A, curve a) vs. Ag/AgCl. Cyclic voltammogram showed anodic shoulders between 1.1 V and 1.35 V due to AuO formation (Au + H_2_O → AuO + 2H^+^ + 2e^−^) and a cathodic peak at 0.95 V in the reverse scan due to the reduction of the oxide films formed [24,25]. When compared with the CVs recorded with Au disc electrode (curve b) and bare PGE (curve c), the 3D crystal formation on PGE elicited larger electroactive response. Under the same conditions, a small residual current was observed in the cyclic voltammogram of the unmodified PGE.

The assembly process of the modified electrode and the electrochemical properties of different modified layers were monitored in 0.1 M KCl containing 5.0 mM [Fe(CN)_6_]^3−/4−^. Cyclic voltammetry is an efficient tool to monitor the kinetic barrier of the interface and the electron transfer between the solution species and the electrode [26]. Figure 3B shows the CVs of bare PGE and CS-IL/PGE and AuNP-CS-IL/PGE, respectively. On the bare PGE (curve a), a characteristic pair of redox peaks of [Fe(CN)_6_]^3−/4−^ couple was observed. The average anodic peak current (Ia) for the bare PGE was 55.12 ± 2.64 µA (*n* = 3). After the immobilization of CS-IL (curve b), peak currents increased (Ia = 78.52 ± 3.72 µA) and peak-to-peak potential difference decreased, indicating a faster electron-transfer could take place at CS-IL interface due to the conductive property of IL. When AuNPs were electrodeposited on this surface (curve c), the magnitude of the peak currents further increased (Ia = 137.2 ± 4.82 µA) and peak-to-peak separation (ΔEp) obviously decreased. This may be attributed to excellent catalytic activity of AuNPs. Moreover, the synergistic effect of composite and the large surface area contributed to the improvement in the conductivity of the AuNP-CS-IL modified electrode. The increase in surface area of the PGE after modifications were calculated using the Randles–Sevcik equation:

ip = (2.69 × 10^5^) n^3/2^ ACD^1/2^ ʋ^1/2^, (1)

where ip is peak current, C is the analyte concentration, n is the number of electrons transferred, ʋ is the rate at which the potential is swept (V s^−1^), D is the diffusion coefficient of the analyte (cm^2^ s^−1^), 7.6 × 10^−6^ cm^2^ s^−1^ (at T = 298 K), and A is the working electrode area (cm^2^). The calculated electroactive surface area of the bare electrode is 0.066 ± 0.003 cm^2^ and AuNP-CS-IL coating on the surface significantly increased the surface area to 0.165 ± 0.006 cm^2^.

Electrochemical impedance spectroscopy (EIS) was used in order to demonstrate the interfacial properties of the electrodes. Figure 3C displays the Nyquist plots of different electrodes with the frequencies ranging from 0.1 Hz to 10 kHz, in which the semicircular portion represents an electron-transfer-limited process at high frequencies. The diameter of the well-defined semicircle in the Nyquist plot corresponding to charge transfer resistance, Rct, decreased, after the introduction of IL-CS and IL-CS-AuNPs on PGE, respectively. The corresponding decrement in the resistance to charge transfer may come from the superior electrical conductivity of ILs and catalytic effect of AuNPs and the increase of the specific surface area of the electrode. Thus, the electron transfer rate increased dramatically (Rct PGE: 5068 ± 440.3 Ω, Rct CS-IL/PGE: 930.6 ± 80.86 Ω, Rct AuNP-CS-IL/PGE: 139.5 ± 12.12 Ω). These results were consistent with the results of the CV measurements mentioned in the previous section similar to the earlier study reported by Safavi et al. [23].

Comparison of cyclic voltammogram and EIS spectrum of AuNP coated PGE in the absence of CS-IL were shown in Figure S1A,B. Figure S1C represents the EIS spectrum of AuNP-CS-IL/PGE distinctively. The average anodic peak was slightly larger and the Rct value was smaller than AuNP-CS-IL/PGE due to agglomeration of bulk AuNPs on the surface rather than nucleation.

### 3.2. Electrochemical Behavior of Malathion on AuNP-CS-IL/PGE

**Figure 4:**
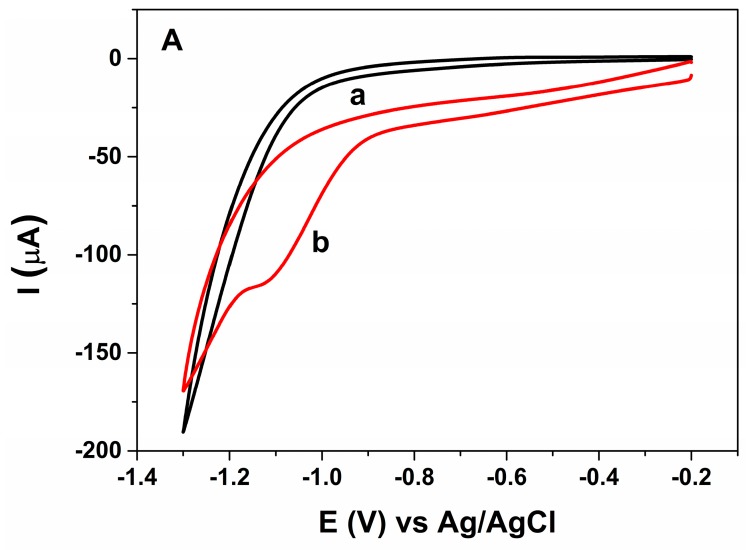

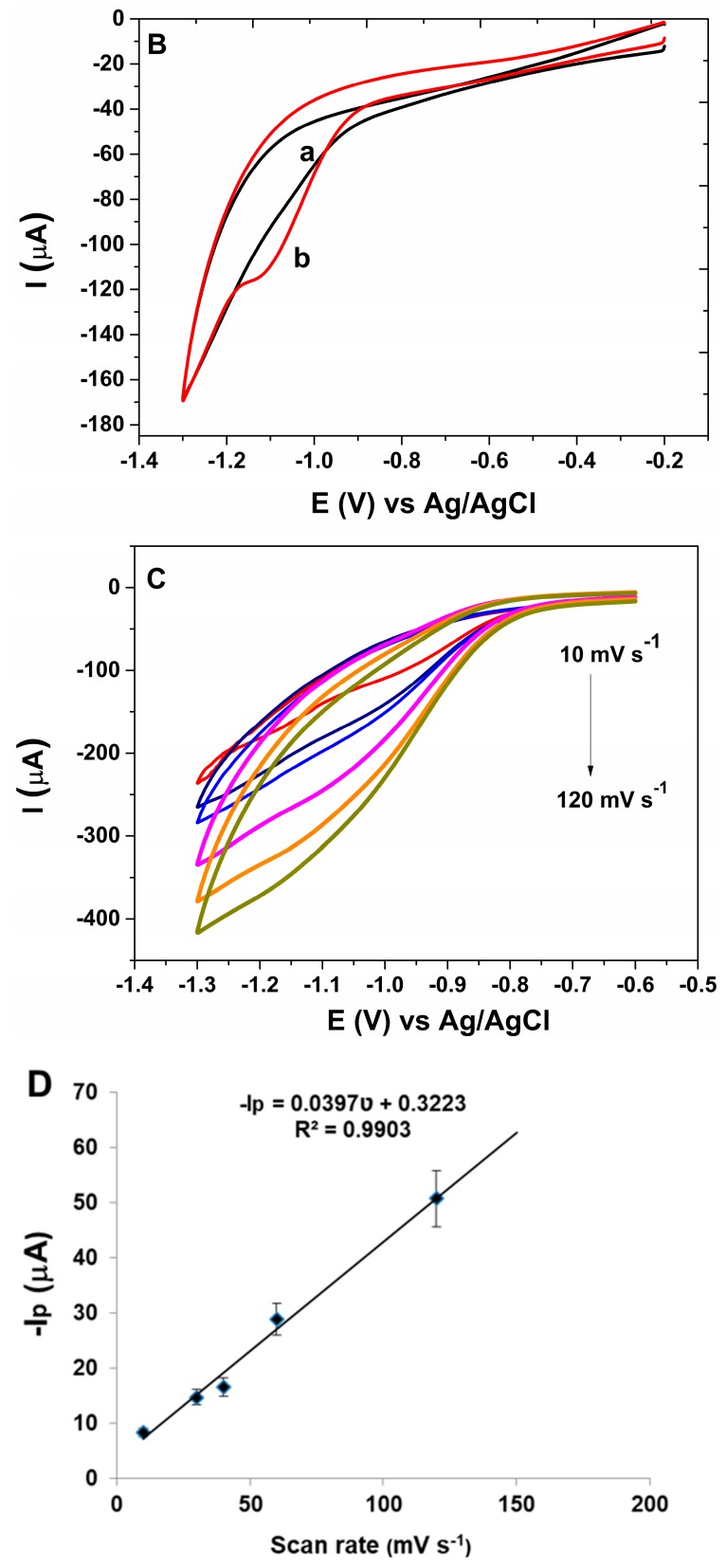

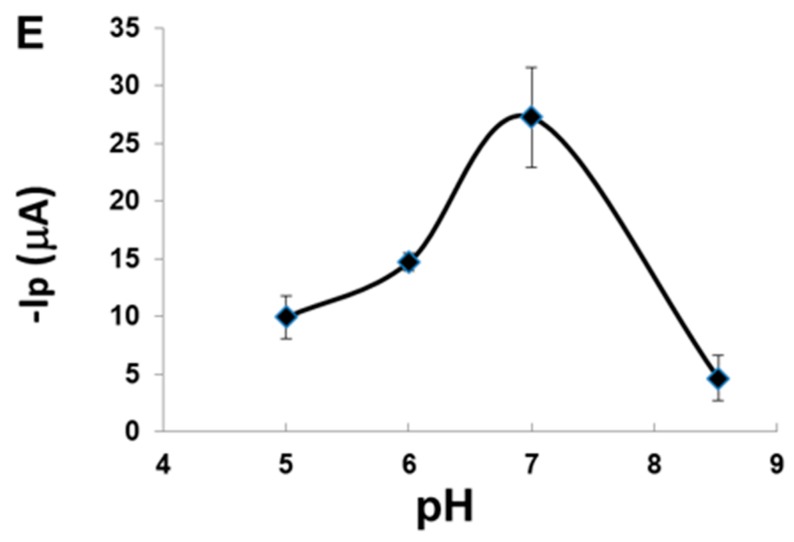
(**A**) cyclic voltammograms of (a) bare PGE (b) AuNP-CS-IL/PGE in pH 7.0 BR containing 20.8 nM MLT. Scan rate: 100 mV s^−1^; (**B**) cyclic voltammograms of AuNP-CS-IL/PGE (a) in the absence (b) in the presence of 20.8 nM MLT in pH 7.0 BR. Scan rate: 100 mV s^−1^; (**C**) cyclic voltammograms of 30 nM MLT (a) AuNP-CS-IL/PGE at scan rates: 10, 30, 40, 60, 80, 120 mV·s^−1^ (shown by arrow); (**D**) the corresponding graph for scan rate vs. peak current; (**E**) effect of pH on MLT signal of AuNP-CS-IL/PGE.

As mentioned, for the electrochemical detection of MLT, the AChE inhibition approach has been most commonly applied by measuring the decrease of the oxidation current of thiocholine, the electroactive product of enzymatic reaction of AChE [4,10,11,12,27]. A few enzymless sensors were constructed that were mostly based on suppression of the current generated by the electrode modifiers in the presence of malathion [3,15]. Thus, in the present work, it was aimed to use AuNP-CS-IL hybrid nanocomposite on PGE to study the direct (non-enzymatic) electrochemical behavior and determination OP pesticide, MLT. The composite electrode was considered to exhibit specific response towards MLT. CV was carried out to study the electrochemical activity of the modified electrode in the absence and the presence of malathion. Figure 4A shows the cyclic voltammograms recorded in pH 7.0 BR buffer solution containing 20.1 nM malathion at the unmodified (curve a) and AuNP-CS-IL (curve b) modified PGE. On the bare electrode, there was no obvious peak under the testing conditions. It is noticeable that at AuNP-CS-IL/PGE, an irreversible reduction peak located at −1.0 V vs. Ag/AgCl corresponding to MLT reduction was observed. This reduction peak was sensitive to the amount of dissolved oxygen in the medium (Figure S2). Therefore, the elimination of the oxygen is important during voltammetric measurements. It should be noted that there may be an interaction between the sulfur atom in the pesticide structure and AuNPs on the sensor. The irreversible reduction peak may be attributed to the two-electron transfer process of the complex formed between malathion and AuNP-CS-IL/PGE [28]:

[Au malathion]^2+^_ads_ + 2e^−^ → Au + Malathion _ads_. (2)

Reduction behavior dithiophosphonates and -P = S- containing structures was also reported in the study of Cekirdek et al. [29], in which the process was examined on a copper deposited glassy carbon electrode. In addition, in the absence of MLT in pH 7.0 BR buffer, there was no peak on AuNP-CS-IL/PGE in the applied voltammetric range (Figure 4B). The results confirmed that the presence of AuNP-CS-IL on PGE has served as a suitable platform for the direct determination of malathion and further increased electron transfer rate on the electrode surface. This excellent detection performance was assigned to the high affinity between AuNP-CS-IL composite film and P = S group of OPs pesticide, malathion.

The effect of the scan rate on the electrochemical behavior of MLT at the AuNP-CS-IL modified pencil graphite electrode was investigated by CV (Figure 4C). There was a linear relationship between reduction peak currents (ip) and scan rates (ʋ) within the scan rate 10–120 mV s^−1^ indicating that the reduction process on this modified electrode was a typical surface (adsorption) controlled process (Figure 4D) [30].

Peak current of MLT was observed in the pH range between 5.0 and 8.5. Peak potential, Ep was observed around −1.0 V vs. Ag/AgCl and did not change significantly with pH. However, the properties of the electrode were influenced by the pH. At lower pH values, possible adsorption of H^+^ on the electrode surface and, at higher pH values, degradation of the OP pesticides may have inhibited the reduction process of MLT (Figure 4E). Maximum peak current was attained at about pH 7.0, so this pH value was chosen as the appropriate value considering the sensitivity.

### 3.3. Analytical Performance of AuNP-CS-IL/PGE towards MLT Determination

**Figure 5:**
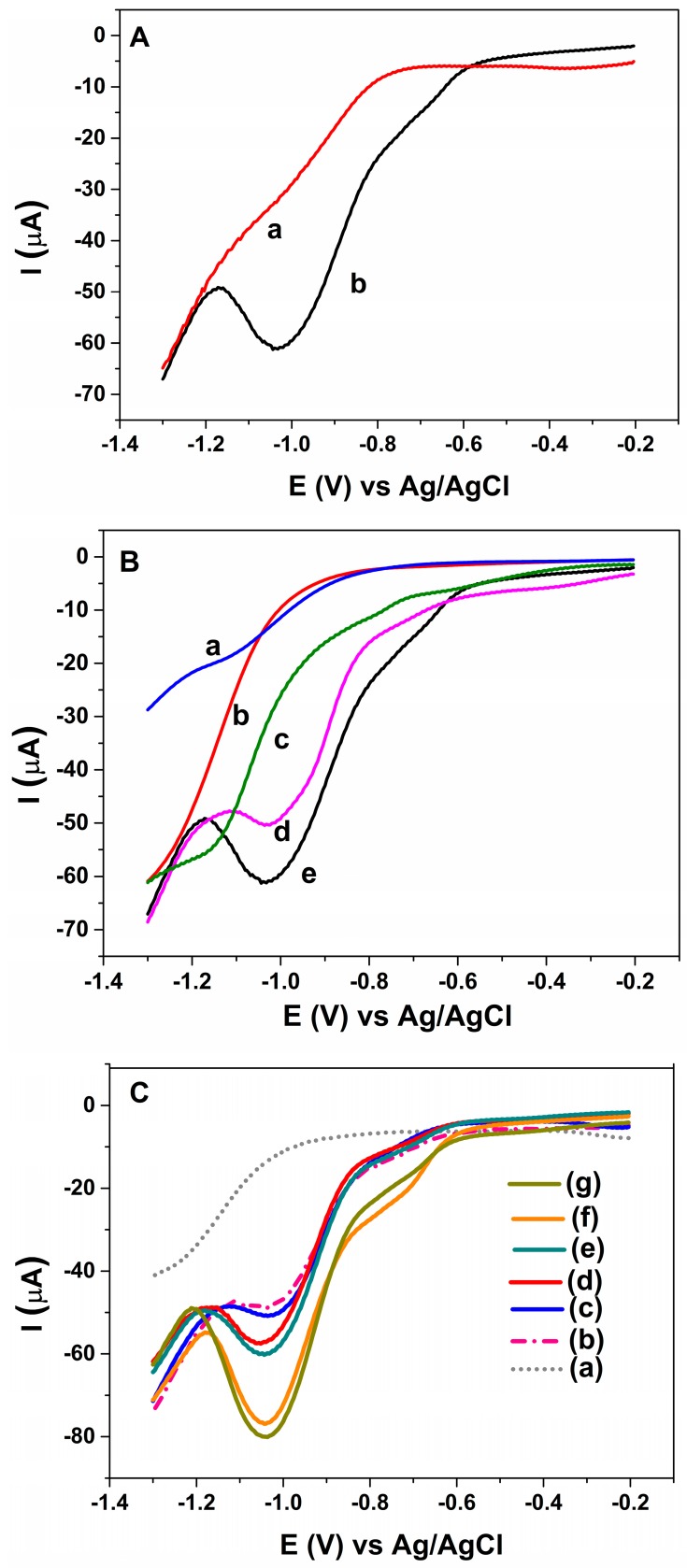

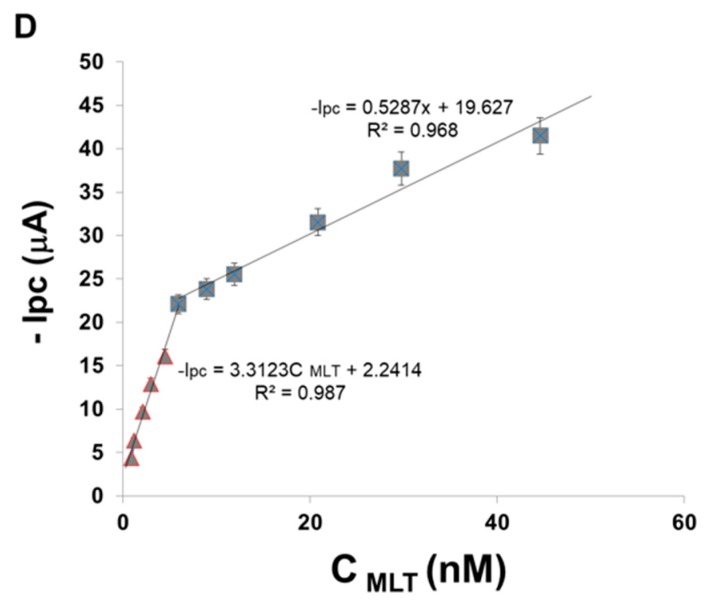
SWVs for (**A**) AuNP-CS-IL/PGE in pH 7.0 BR buffer containing (a) 0 nM (b) 3 nM MLT; (**B**) (a) bare Au disc electrode (b) bare PGE (c) CS-IL/PGE (d) AuNP/PGE (e) AuNP-CS-IL/PGE in pH 7.0 BR buffer containing 3 nM MLT (Accumulation time: 300 s); (**C**) SWVs of MLT at AuNP-CS-IL/PGE in pH 7.0 increasing concentrations of MLT (from a to g): 0.0; 2.97; 8.9; 14.9; 20.8; 44.5; 50.0 nM; (**D**) calibration graph corresponding to MLT concentrations vs. peak currents (Accumulation time: 300 s).

The effect of modification on the determination of MLT was investigated by using a square wave voltammetry (SWV) technique, which yields higher sensitivity in electrochemical measurements. After accumulation of MLT on the sensor surface by stirring the solution for 300 s under open-circuit, the square wave voltammogram for the electroreduction of 3.0 nM MLT in BR buffer (pH = 7.0) at the AuNP-CS-IL/PGE was examined in Figure 5A. SWV response of the adsorbed MLT exhibited a well-defined reduction peak around −1.0 V vs. Ag/AgCl (curve b). In contrast, SWV signals for MLT at unmodified PGE, CS-IL/PGE, AuNP/PGE or Au disc electrode were weak over the same conditions (Figure 5B). This observation strongly demonstrated the importance of combination of electrochemically synthesized AuNPs possessing catalytic activities because of their quantum size effect [31] with good adsorbing and electrical properties of CS-IL in MLT sensing. The results showed that the presence of CS, ILs and AuNPs together increased the effective surface area and favoured excellent electrochemical performance for the non-enzymatic MLT determination. The interaction between malathion with AuNP-CS-IL/PGE was strong enough that the regeneration of the sensor surface was not possible. Thus, the electrode surface is designed as single use and disposable.

Figure 5C shows the SWV curves for different concentrations of MLT on AuNP-CS-IL/PGE. The peak currents located around −1.0 V vs. Ag/AgCl corresponding to MLT reduction increased with MLT concentration, displaying two different concentration ranges depending upon high and low affinity due to the remarkable adsorptive capability of the sensor (Figure 5D). Depending on the high number of active binding sites on the electrode surface for lower MLT concentration, the slope of the first linear region of calibration curve is high, whereas higher concentrations of MLT yielded a decreased slope of the calibration curve, which may reasonably be due to the kinetic limitation caused by decreased binding sites of the electrode surface. The linear equations were: −Ipc (μA) = 3.312 C (nM) + 2.2414 (R^2^ = 0.987) in the range of 0.89–5.94 nM and −Ipc (μA) = 0.5287 C (nM) + 19.657 (R^2^ = 0.968) in the range of 5.94–44.6 nM, respectively with a detection limit (LOD) of 0.68 nM. The LOD was calculated from 3 S_b_/m, where S_b_ is the standard deviation of blank and m is the slope of the regression line.

The comparison of the performance of the AuNP-CS-IL modified electrode with other reports based on enzyme inhibition and nanostructured materials is shown in Table 1. The detection limit estimated in this work is relatively low and comparable with the literature using enzymes and other types of modified electrodes.

The influence of possible interfering substances on the determination of MLT was studied where the concentration of MLT was fixed at 15 nM. The electrode response was examined for 1000-fold higher quantity of an organophosphorus pesticide, fenitrothion and 2 mM of some potential interfering ions in water. The results were displayed in Table 2. The peak current was not significantly affected by the substances, so AuNP-CS-IL modified electrode was highly selective to MLT.

To evaluate the suitability of the biosensor for real samples, recovery studies were carried out. Tomato and apple samples were spiked with different standard concentrations of MLT. The recoveries in the range of 91.1–114.2% were summarized in Table 3, demonstrating the accuracy of AuNP-CS-IL/PGE sensor.

## 4. Conclusions

PGEs were easily modified with an AuNP-CS-IL hybrid to detect an organophosphorus pesticide, malathion directly, for the first time. The benefits of ILs (high conductivity and good adsorption for OPs), CS (biocompability, good film forming ability and) and AuNPs (high electrocatalytic activity, good adsorption capability for thio containing groups) were combined on disposable pencil graphite electrodes in a synergistic effect. The nanocomposite film not only enhanced the surface area of the electrodes but also provided an excellent platform for obtaining the electrochemical response of malathion without the assistance of AChE. Square wave voltammetry has been applied in order to investigate the electrochemical performance of the AuNP-CS-IL/PGE based sensor towards malathion. The presented sensor was a promising sensing platform for the highly sensitive and fast determination of malathion, which is important for public health, environment environmental security and food safety. The proposed non-enzymatic approach was simple, efficient and cost-effective for the analysis of MLT in food samples and this method can be used as an alternative in the future.

## Figures and Tables

**Table 1:** Analytical characteristics of different electrochemical biosensors for the determination of MLT.

Electrode	MLT Linear Range	Electrochemical Technique	LOD	Reference
AChE–AuNPs–CaCO_3_–Au–SiSG	0.1–100 nM	CV	0.1 nM	[27]
AChE-Fe_3_O_4_NP-MWCNTs/Au	0.1–40 nM	Amperometry	0.1 nM	[12]
AChE–SiSG–CPE	0.07–1.3 ppm	DPV	0.058 ppm (0.174 nM)	[4]
CS/AChE/PB-CS/ERGO-AuNPs-β-CD/GCE	7.98–2 × 10^3^ pg mL^−1^	Amperometry	4.14 pg mL^−1^	[11]
Poly(TTP)/AChE/GCE	9.99–99.01 nM	CV	4.08 nM	[10]
CuO NWs–SWCNTs/GCE	0.3–1.4 nM	DPV	0.3 nM	[15]
Gly-CuO/GCE/nafion	1–12 nM	DPV	0.1 nM	[16]
PANI-Nanofibers-SWCNTs Graphite Electrode	2 × 10^−7^–14 × 10^−7^ mol L^−1^	DPV	2 × 10^−7^ mol L^−1^	[3]
Mitochondria modified paper based electrodes	20–60 nM	CV	20 nM	[32]
**AuNP-CS-IL/PGE**	**0.89–5.94 nM and 5.94–44.6 nM**	**SWV**	**0.68 nM**	**Present work**

AChE—Acetylcholinesterase; AuNPs—Au nanoparticles; CaCO_3_—calcium carbonate; SiSG—silica sol–gel; Fe_3_O_4_NP—iron oxide nanoparticle; Au—gold electrode; MWCNTs—multiwalled carbon nanotubes; CPE—carbon paste electrode; PANI—polyaniline; PB-CS—Prussian blue-chitosan; ERGO—reduced graphene oxide; β-CD—β-cyclodextrin; GCE—glassy carbon electrode; Poly(TTP)—poly([2,2′;5′,2″]-terthiophene-3-carbaldehyde; CuO NWs—copper oxide nanowires; Gly-CuO—glycine-copper oxide nanowires; CV—cyclic voltammetry; SWV—square wave voltammetry; DPV—differential pulse voltammetry.

**Table 2:** The effect of some interferents on MLT signal. **^a^** Current ratio = I_H+I_/I_H_. I_H+I_, MLT signal in the presence of interfering species. I_H_, MLT (15 nM) signal.

Interfering Species	Current Ratio ^a^
K^+^	1.020
Na^+^	0.990
Bi^3+^	0.900
SO_4_^2−^	1.101
NO_3_^−^	0.900
Cl^−^	0.950
Fenitrothion	0.986

**Table 3:** Recovery tests of MLT in tomato and apple samples (n = 3).

Sample	Malathion Added (nM)	Malathion Found (nM)	Recovery (%)	RSD (%, n = 3)
Tomato	0.893	1.02	114.2	7.9
	11.01	10.1	91.7	10.5
Apple	0.893	0.814	91.1	2.3
	1.488	1.570	105.5	10.4

## Supplementary Materials

The following are available online at http://www.mdpi.com/1424-8220/18/3/773/s1, Figure S1: (A) CVs of a) Bare PGE b) CS-IL/PGE c) AuNP-CS-IL/PGE d) AuNP/PGE in 0.1 M KCl containing 5.0 mM [Fe(CN)_6_]^3−/4−^. Scan rate: 50 mV s^−1^. (B) Electrochemical impedance spectroscopy (EIS) of a) Bare PGE, b) CS-IL/PGE, c) AuNP-CS-IL/PGE, d) AuNP/PGE in 0.1 M KCl containing 5.0 mM [Fe(CN)_6_]^3−/4−^ (C) EIS of AuNP-CS-IL/PGE in 0.1 M KCl containing 5.0 mM [Fe(CN)_6_]^3−/4−^, (Frequency range 0.1-100,000 Hz). Figure S2: AuNP-CS-IL/PGE in pH 7.0 BR containing 20.8 nM MLT a) in the presence of oxygen, b) nitrogen gas purged. Scan rate: 100 mV·s^−1^.
