# Supplementary material for: Non-Enzymatic Electrochemical Sensing of Malathion Pesticide in Tomato and Apple Samples Based on Gold Nanoparticles-Chitosan-Ionic Liquid Hybrid Nanocomposite
Source: Sensors (Basel). 2018 Mar 5;18(3):773. doi: 10.3390/s18030773 (PMC5876763; doi:10.3390/s18030773)
Supplement: Supplementary file 1 [file sensors-18-00773-s001.pdf]

Non-enzymatic electrochemical sensing of malathion pesticide in tomato and apple samples based on gold nanoparticles-chitosan-ionic liquid hybrid nanocomposite

Gulcin Bolat, Serdar Abaci *

Hacettepe University, Faculty of Science, Department of Chemistry, 06800, Beytepe-Ankara, Turkey;
e-mails: gbolat@hacettepe.edu.tr; sabaci@hacettepe.edu.tr

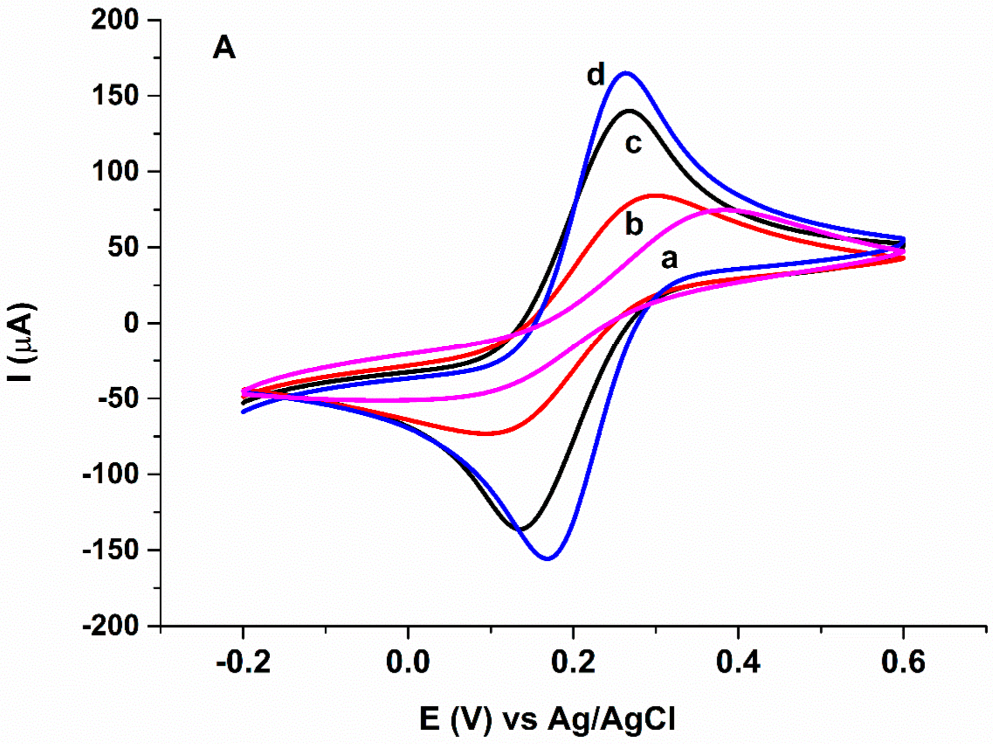

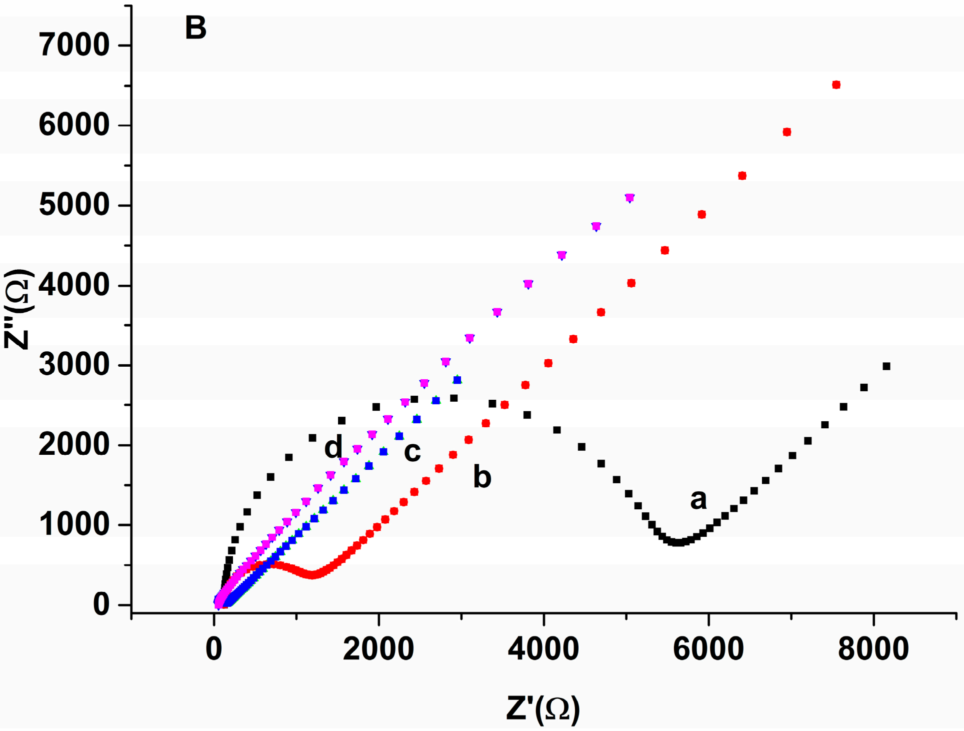
B
7000
6000
5000
4000
3000
2000
1000
0
Z''(Ω)
0
2000
4000
6000
8000
Z'(Ω)
a
b
c
d

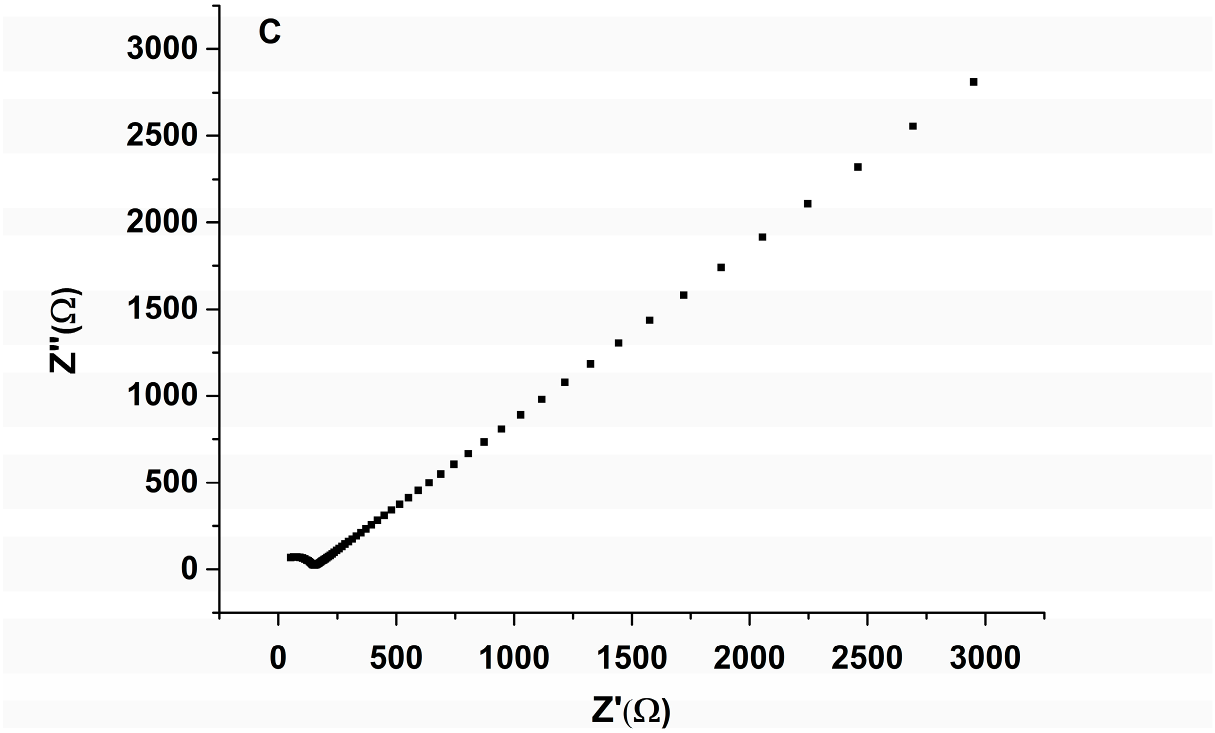

Figure S1. (A) CVs of a) Bare PGE b) CS-IL/PGE c) AuNP-CS-IL/PGE d) AuNP/PGE in 0.1 M KCl containing 5.0 mM $[Fe(CN)_6]^{3-/4-}$. Scan rate: 50 mV s^{-1}. (B) Electrochemical impedance spectroscopy (EIS) of a) Bare PGE, b) CS-IL/PGE, c) AuNP-CS-IL/PGE, d) AuNP/PGE in 0.1 M KCl containing 5.0 mM $[Fe(CN)_6]^{3-/4-}$ (C) EIS of AuNP-CS-IL/PGE in 0.1 M KCl containing 5.0 mM $[Fe(CN)_6]^{3-/4-}$, (Frequency range 0.1-100,000 Hz).

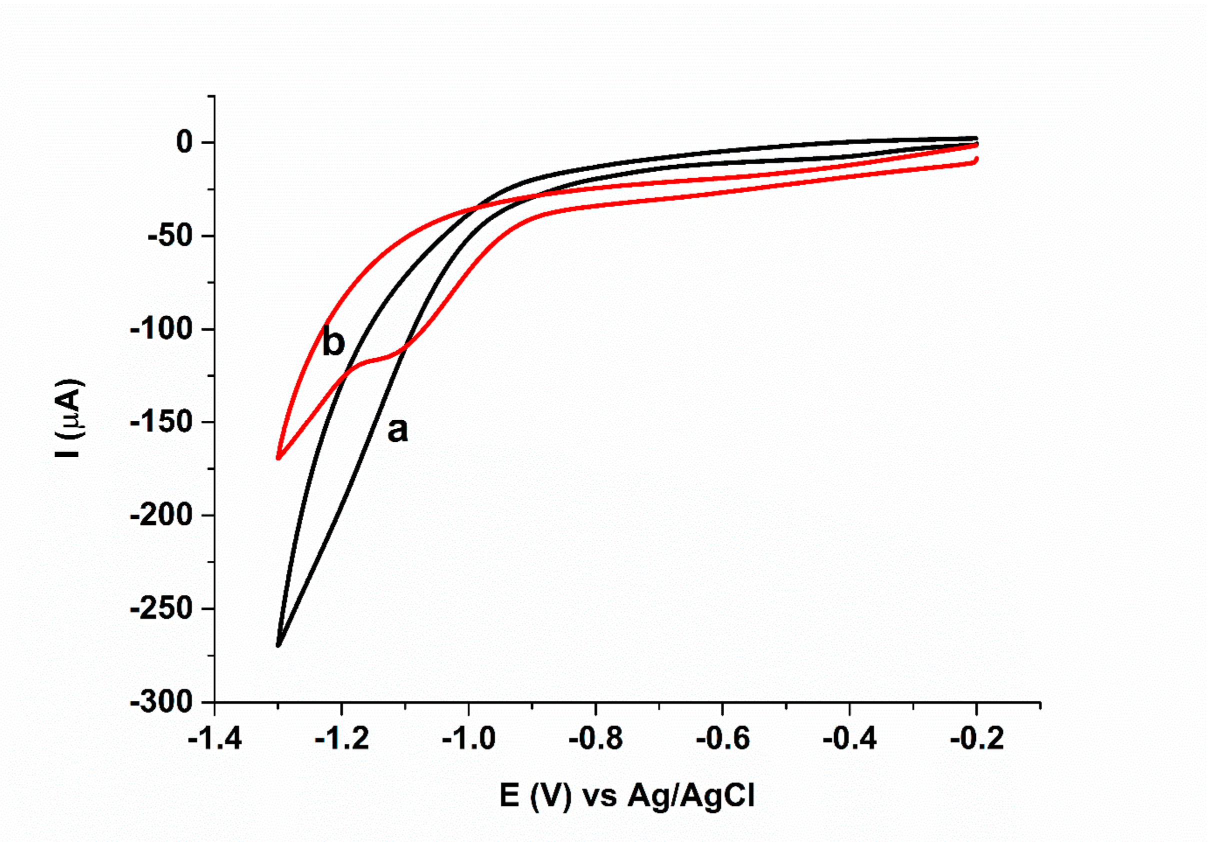

Figure S2. AuNP-CS-IL/PGE in pH 7.0 BR containing 20.8 nM MLT a) in the presence of oxygen, b) nitrogen gas purged. Scan rate: 100 mV.s^{-1}.
